# Results of Operations on Scrofulous Persons

**Published:** 1876-04

**Authors:** 


The Results of Operations on Scrofulous Persons.—M. Bernard, in the course of his studies on scrofula in general, has arrived at the following conclusions on the subject. In the scrofulous the grave complications which succeed the traumatism are rare. The attacks of erysipelas or of lymphangitis are mild, when they occur at all, while diffuse cellulitis, generous inflammation, and pyæmia are hardly ever met with. In general the scrofulous resist well the shock of an operation, so much so that Gerdy has been able to cite three amputations for white swelling in which the traumatic fever was almost nil. But it must be confessed that, although the scrofulous support the shock of an operation well, and rarely succumb during the first two or three weeks, the period of danger lasts much longer in them, and the final result is much more doubtful. In numerous cases the operation produces both local alleviation and an amelioration of the general symptoms, by removing a constant source of pain and exhaustion ; but this temporary improvement, though raising hopes of a complete recovery, does not persist, and the patient soon after dies, worn out by the general disease. M. Verneuil expressed himself as follows on this subject : "Surgical intervention rarely arrests scrofula, aggravates it sometimes, but most frequently does not affect its evolution at all." M. Bernard speaks strongly of the value of alteratives, and especially of sea baths and country air.—*Le Bordeaux Medical*, January 25, 1876.

